# Late effect of larval co-exposure to the insecticide clothianidin and fungicide pyraclostrobin in Africanized *Apis mellifera*

**DOI:** 10.1038/s41598-019-39383-z

**Published:** 2019-03-01

**Authors:** Rafaela Tadei, Caio E. C. Domingues, José Bruno Malaquias, Erasnilson Vieira Camilo, Osmar Malaspina, Elaine C. M. Silva-Zacarin

**Affiliations:** 1 Universidade Federal de São Carlos (UFSCar), Campus Sorocaba, Programa de Pós-Graduação em Biotecnologia e Monitoramento Ambiental, CCTS. Rodovia João Leme dos Santos km 110, Itinga, 18052-780 Sorocaba, SP Brazil; 2 Universidade Federal de São Carlos (UFSCar), Campus Sorocaba, Departamento de Biologia, CCHB. Rodovia João Leme dos Santos km 110, Itinga, 18052-780 Sorocaba, SP Brazil; 3 Universidade Estadual Paulista (UNESP) – “Júlio de Mesquita Filho”, Campus Rio Claro, Programa de Pós-Graduação em Biologia Celular e Molecular. Av. 24 A, 1515, Jardim Bela Vista, 13506-900 Rio Claro, SP Brazil; 4 Department of Entomology and Acarology, Luiz de Queiroz College of Agriculture (ESALQ), University of São Paulo (USP), Av. Pádua Dias 11, Piracicaba, 13418-900 São Paulo Brazil; 5 Department of Agricultural Statistics and Experimentation, Luiz de Queiroz College of Agriculture (ESALQ), University of São Paulo (USP), Av. Pádua Dias 11, Piracicaba, 13418-900 São Paulo Brazil

## Abstract

Among the factors that contribute to the reduction of honeybee populations are the pesticides. These chemical compounds reach the hive through forager bees, and once there, they can be ingested by the larvae. We evaluated the effects of repeated larval exposure to neonicotinoid insecticide, both in isolation and in combination with strobilurin fungicide, at environmentally relevant doses. The total consumption of the contaminated diet was 23.63 ng fungicide/larvae (pyraclostrobin) and 0.2364 ng insecticide/larvae (clothianidin). The effects on post-embryonic development were evaluated over time. Additionally, we assessed the survival pattern of worker bees after emergence, and the pesticides’ effects on the behavior of newly emerged workers and young workers. Young bees that were exposed to the fungicide and those subjected to co-exposure to both pesticides during larval phase showed behavioral changes. The insecticide, both in isolation and in combination with fungicide reduced the bees’ longevity; this effect of larval exposure to pesticides was stronger in bees that were exposed only to the insecticide. Although the larvae did not have sensitivity to exposure to pesticides, they showed later effects after emergence, which may compromise the dynamics of the colony, contributing to the reduction of the populations of bees in agroecosystems.

## Introduction

Bees are insects that play an important and beneficial role in the pollination of plants of economic interest for food production, as well as performing pollination services that maintain plant biodiversity in different biomes and landscapes^1,2^. However, the decline in the number of honeybee colonies has been observed for some years in the USA, Europe and Brazil^3–6^.

Evidence for the increasing declines in bee populations points to a multitude of biotic and abiotic factors^7,8^, including exposure to pesticides, pathogens and parasites, as well as environmental factors such as habitat loss, fragmentation, and nutritional restrictions due to the expansion of monocultures^6,9–11^.

Bees are often exposed to pesticides by feeding on potentially contaminated nectar and pollen which is collected in the agricultural crops by the forager workers and transported to the colony, where it is used as food for all individuals, including larvae that are fed by the nurse bees^12^. Workers’ larvae are fed with bee bread (mixture of secretion of mandibular and hypopharyngeal glands containing small amounts of pollen and nectar)^13–15^ and they can be exposed to the pesticides either orally or by contact during the feeding phase^12^, as potentially contaminated food is deposited by the worker bees inside the brood cells. Despite the larvae’s intense food intake, it does not eliminate the excreta until the end of the larval development^16^, bioaccumulating the xenobiotics until the beginning of its metamorphosis, which is marked by defecation, when the midgut connects to the posterior intestine and empties its content^17^.

A wide variety of pesticides have been found in plant pollen. Among them are fungicides such as pyraclostrobin, and the neonicotinoid class of insecticides such as clothianidin, which are characterized by their systemic actions on plants and their persistence and mobility in the environment^2,4,8,18–24^. Pesticides of systemic action are translocated in the plant and thus reach pollen and nectar, representing a potential risk for pollinators^25,26^.

The effects of neonicotinoids are widely studied in *Apis mellifera* by the use of various toxicological parameters^27–33^. However, toxicological data on the exposure of these bees to fungicides are rare, both in isolation and in combination with neonicotinoids^34–37^, and the little data available refers only to adult worker bees^38^.

Studies have shown that these pesticides on their own can cause decline of *A*. *mellifera* colonies^36,39,40^, but in realistic field conditions chemical compounds are not isolated, but a mixture^22,41–48^. The combination of insecticides and fungicides may further increase their toxicity and, consequently, have stronger deleterious effects on the colonies^4,11,45,49,50^.

Less than 20% of all studies related to exposure of honeybees to pesticides use larvae in their methodology, and only 6.3% of all studies report on the combination of insecticides and fungicides^51^. Given the current scenario of declining populations of bees, threatening food production, gaps in effects of combined pesticides^10,15,49,50,52^, and the scarcity of studies on larval toxicity as evidenced by Simon-Delso *et al*.^9^, the present study aims to add information relevant to the understanding of the effects of exposure to pesticides on bee larvae^1,2,53^. Additionally, this research provides information regarding the effects of larval exposure to a combination of insecticides and fungicides during post-embryonic development, and evaluates these effects on subsequent adults.

The objective of this study is to evaluate the effects of repeated oral exposure to both clothianidin (neonicotinoid insecticide) and pyraclostrobin (strobilurin fungicide), in isolation and in combination, in worker larvae of Africanized *A*. *mellifera*. These effects were evaluated by means of different biological parameters in larvae, pupae and emerged adults.

## Results

### Evaluation of effects throughout development

There was no significant difference between the larval mortality in the control and solvent control (acetone) groups, pyraclostrobin fungicide, clothianidin insecticide and the combination of pesticides (clothianidin + pyraclostrobin) (*P* > 0.9), and in all of these groups, accumulated mortality from the forth (D4) to the seventh day (D7) did not exceed 12% (Table 1). The larvae exposed to dimethoate showed a mean mortality of 94.01%, validating the bioassay, according to OECD protocol n° 239. Exposure to pesticides during the larval phase did not cause delay in development.

**Table 1:** Mortality (means ± standard error) of larval and pupal phase, pupation and emergence rate in Africanized *A*. *mellifera*.

Experimental group	Average mortality		Rate (%)	
	Larval (%)	Pupal (%)	Pupation	Emergence
Control	9.38 ± 0.73	13 ± 2.0	93 ± 0.83	77.20 ± 1.07
Solvent control	10.42 ± 0.82	12 ± 1.4	97 ± 0.23	84.18 ± 0.42
Clothianidin Insecticide	9.47 ± 0.54	21 ± 2.71	87 ± 0.91	86.23 ± 0.74
Pyraclostrobin Fungicide	11.46 ± 0.81	19 ± 1.96	88 ± 0.88	86.25 ± 0.37
Co-exposure	10.42 ± 0.25	11 ± 1.53	93 ± 0.27	88.73 ± 1.24
*χ* ^2^	=0.98957	—	=0.1836	—
*F*	—	=0.7410	—	=0.2608

*χ* = Chi squared value.

*F* = *F* value.

The experimental groups did not differ in terms of pupal mortality (*P* > 0.7), pupation rate (*P* > 0.1) and emergence rate (*P* > 0.2) (Table 1). The mean time of emergence in the groups was 19 days from the grafting of the instar larvae.

### Behavioral analysis

**Figure 1:**
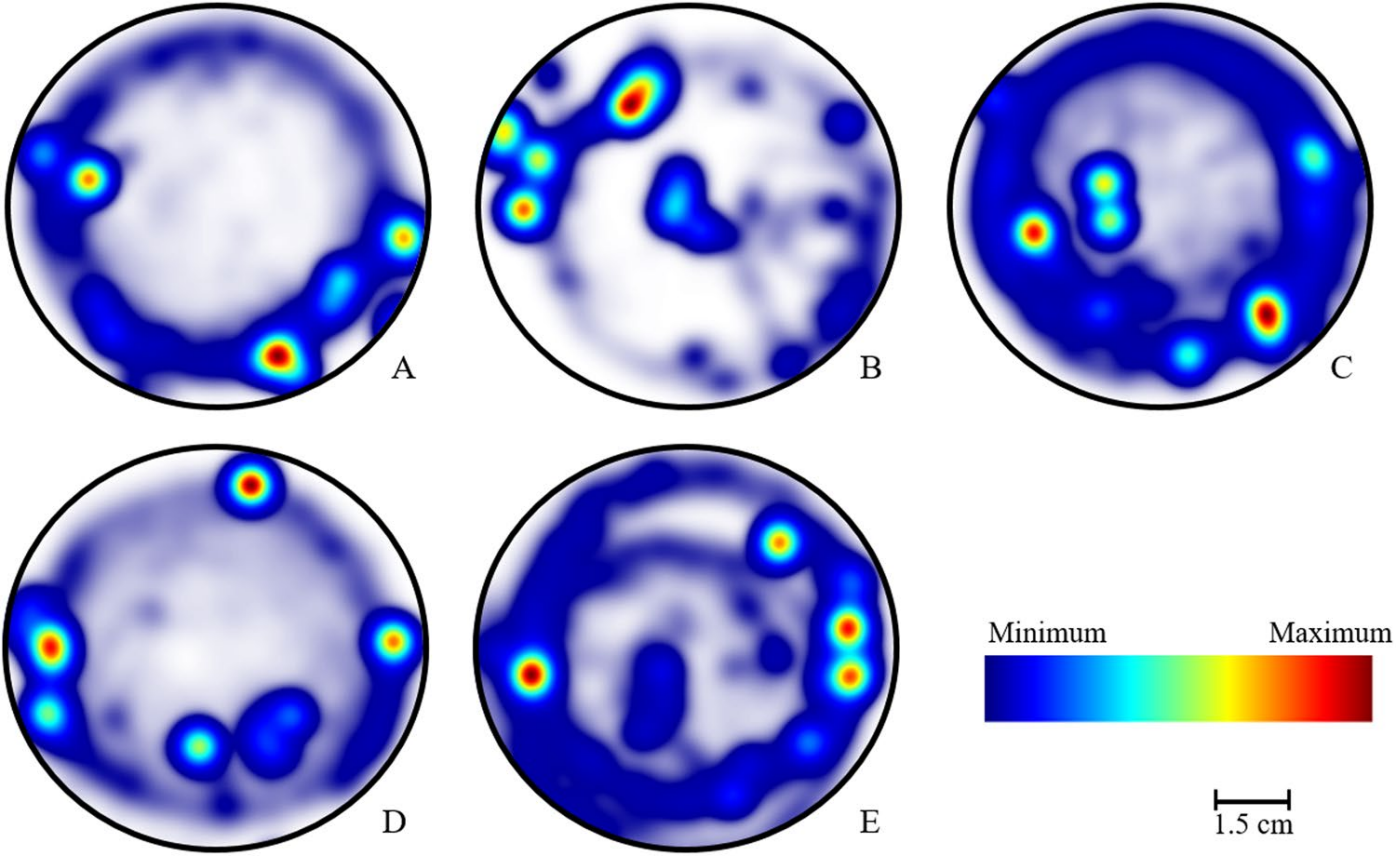
Ethogram of newly emerged Africanized *A*. *mellifera* at 1 day old (N = 7). Walking pattern of bees and frequency of their position in arena over time (t = 10 min). (**A**) Control; (**B**) Solvent control; (**C**) Clothianidin Insecticide; (**D**) Pyraclostrobin Fungicide; (**E**) Co-exposure (Insecticide + Fungicide).

The ethogram of newly emerged bees (Fig. 1) showed a pattern of movement and occupation of the arena that was similar in the experimental groups. In this pattern, the bees remained immobile for longer periods of time and, when they moved, their movements were circular around the arena. However, bees exposed to clothianidin, as well as those exposed to the two combined pesticides, had a wider occupancy pattern in than in other treatments.

**Figure 2:**
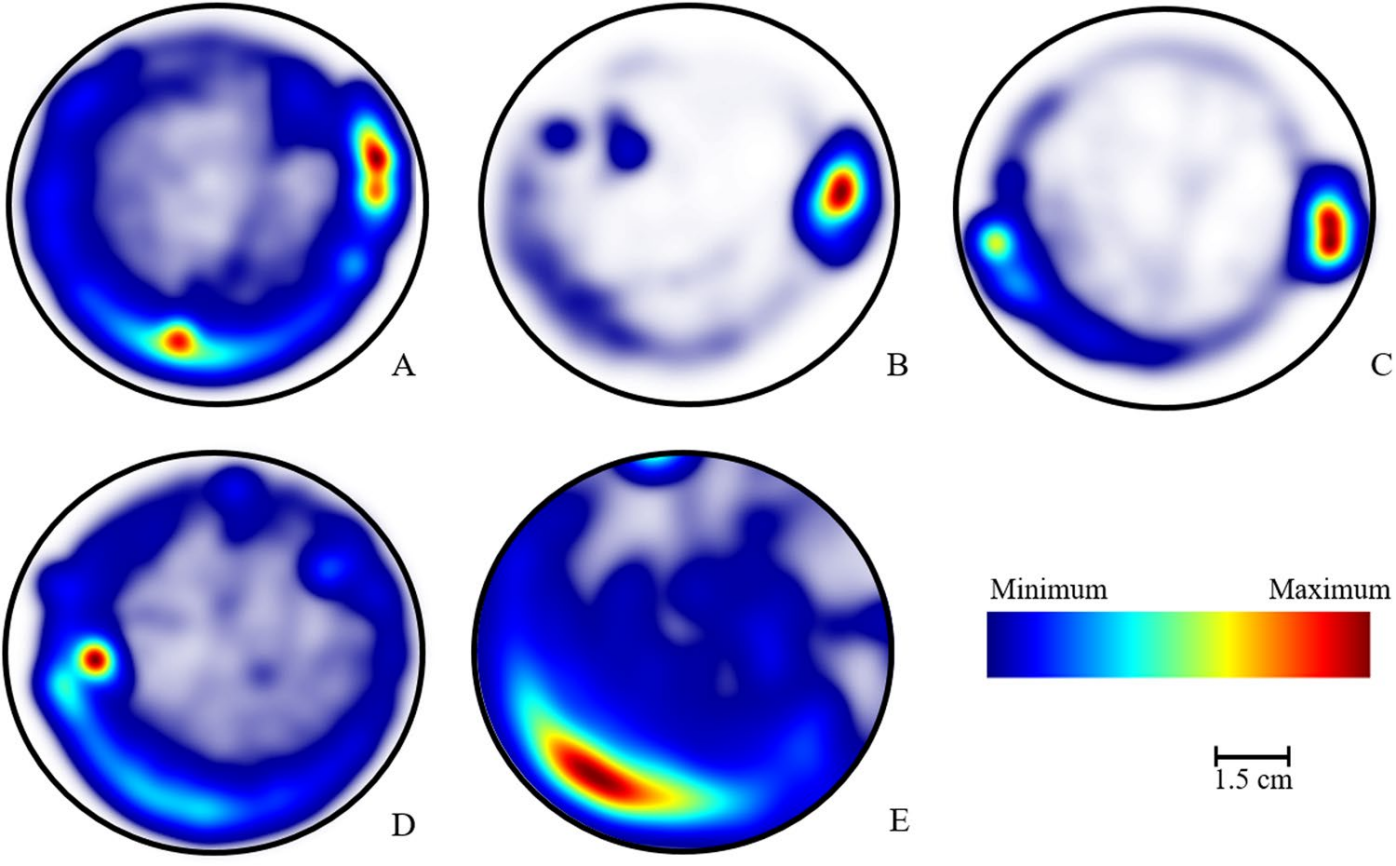
Ethogram of young Africanized *A*. *mellifera* at 3–4 days old (N = 7). Walking pattern of bees and frequency of their position in arena over time (t = 10 min). (**A**) Control; (**B**) Solvent control; (**C**) Clothianidin Insecticide; (**D**) Pyraclostrobin Fungicide; (**E**) Co-exposure (Insecticide + Fungicide).

The ethogram of young bees (from 3 to 4 days old) exposed to the two combined pesticides (Fig. 2E) showed an occupancy pattern in a larger area of the arena, but the circular movement of these bees was not evident in the Petri dish. In the insecticide exposed-group and solvent control-group, the occupation of bees was concentrated to some points of the arena.

**Figure 3:**
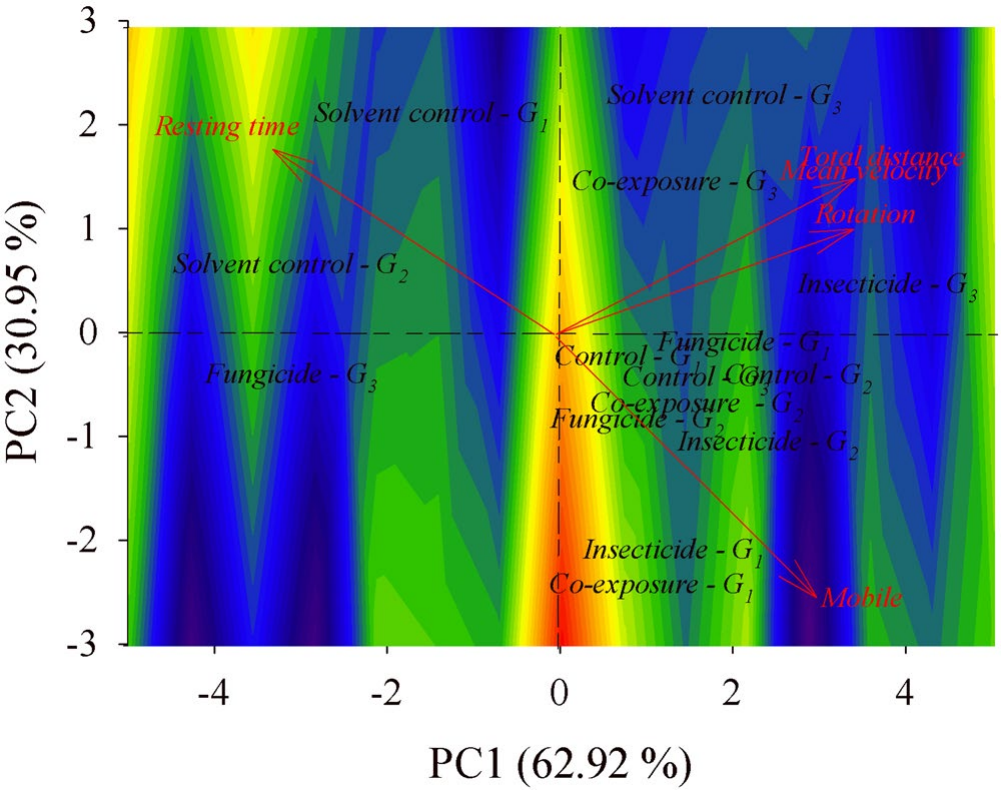
Biplots for the of newly emerged *A*. *mellifera* from control (with water or solvent), clothianidin- or pyraclostrobin-exposed groups, and co-exposure to the insecticide and fungicide. The contour area was defined by eigenvectors values map. Each colony (*G*) is exposed in the biplot, totalizing 3 colonies (*G*_1_, *G*_2_, and *G*_3_).

The arrangement of vectors in the biplots (Fig. 3) corresponding to newly emerged adults showed a higher correlation [cosine (*θ*)] between the following variables: resting time and mobile time [cosine (*θ*) = −0.9200], rotation and mobile time [cosine (*θ*) = −0.8100], and rotation and mean velocity [cosine (*θ*) = −0.8400]. There was maximum positive correlation [cosine (*θ*) = 1.0000] involving total distance moved with mean velocity in this age group.

In both ages, two components were selected based on the presence of components with eigenvalues greater than 1 in the correlation matrix. The two components explain, beyond the cumulative sum of the variance in both age groups, more than 90% of total data variation.

According to eigenvector (*v*) values, the first biplot component of newly emerged insects is mainly represented by the weighted average of rotation [*v* = 0.4963] and mobility [*v* = 0.4626]. The first component in this case explains 62.92% of the total data variation. Our data suggests variability in terms of insect co-exposure to the pesticides, as the observation groups of each treatment were not found in the same biplot quadrant.

**Figure 4:**
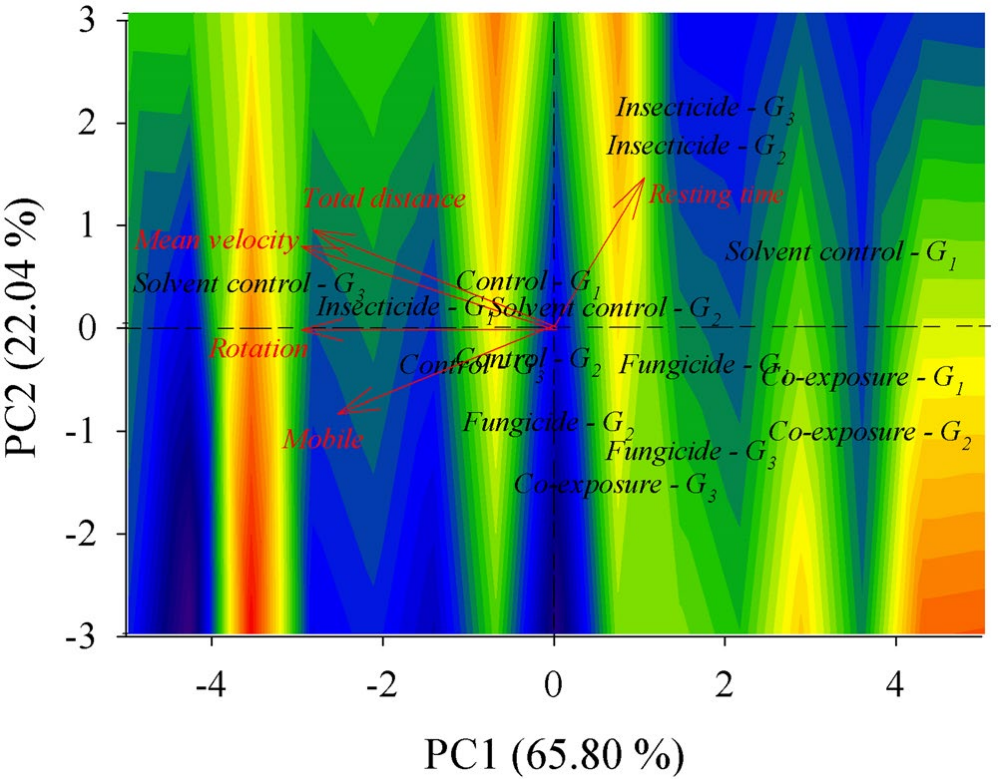
Biplots for the *A*. *mellifera* with 3–4 days old from control (with water or solvent), clothianidin- or pyraclostrobin-exposed groups, and co-exposured to the insecticide and fungicide. The contour area was defined by the eigenvectors values map. Each colony (*G*) is exposed in the biplot, totalizing 3 colonies (*G*_1_, *G*_2_, and *G*_3_).

In relation to worker honeybees at 3–4 days old, we also observed a maximum positive correlation [cosine (*θ*) = 1.0000] involving total distance moved with mean velocity (Fig. 4). At 3–4 days of age, the correlations observed between mobile time and the other variables were: resting time [cosine (*θ*) = −0.8087], total distance [cosine (*θ*) = 0.9626], mean velocity [cosine (*θ*) = 0.9610] and rotation [cosine (*θ*) = 0.9818]. In addition to the negative correlation involving the period of resisting and mobility, resting time was also negatively correlated with total distance [cosine (*θ*) = −0.9245], mean velocity [cosine (*θ*) = −0.9226] and rotation [cosine (*θ*) = −0.8817]. A positive correlation was evidenced between rotation and total distance [cosine (*θ*) = 0.9711] and between rotation and mean velocity [cosine (*θ*) = 0.9701].

The first biplot component of honeybees at 3–4 days old is performed by calculating the weighted average of mobile time [*v* = −0.4830], total distance [*v* = −0.5058], mean velocity [*v* = −0.5082] and rotation [*v* = −0.4803, and the second component largely contributes to the variable resting time [*v* = 0.8835]. The first and second components explain the data variations of 65.80 and 22.04%, respectively.

High variability was also observed in bees that were 3–4 days old in all experimental groups. However, it was possible to highlight that when honeybees were exposed to the fungicide or submitted to co-exposure (fungicide and insecticide), there was an evident trend of decreased total distance moved and mean velocity when compared to other treatments. Due to the observations of both treatments (fungicide and co-exposure to both pesticides), they were placed spatially close to each other on the biplot and away from mean velocity and total distance vectors.

The behavior of the newly emerged bees did not differ significantly between the control and solvent control groups (Table 2). In comparison to the solvent control group, bees exposed to clothianidin and co-exposed to both pesticides presented higher mobile time and, in addition, clothianidin-exposed bees had lower resting time (Table 2). The other variables did not present differences between the experimental groups.

**Table 2:** Ingestion effects of pesticides-contaminated diet on the following behavioral variables (means ± standard error): mobile time, resting time, total moved distance, mean velocity, frequency of rotation and period of high mobility in newly emerged honeybees (*A*. *mellifera*).

Group	Mobile (s)	Resting time (s)	Total distance (cm)^1^	Mean Velocity (cm/s)^1^	Rotation (frequency)^1^	High mobility (s)^1^
Control	121.05 ± 56.88 ab	6.70 ± 2.32 ab	963.06 ± 334.75	1.67 ± 0.53	11 ± 3.56	0.32 ± 0.03
Solvent control	22.16 ± 12.43 b	16.47 ± 4.97 a	996.34 ± 671.81	1.64 ± 1.10	5.86 ± 4.41	0.21 ± 0.06
Clothianidin Insecticide	185.48 ± 63.31 a	5.59 ± 2.89 b	1081.32 ± 384.65	1.74 ± 0.59	10.43 ± 5.32	0.3 ± 0.02
Pyraclostrobin Fungicide	103.91 ± 45.78 ab	8.80 ± 2.9 ab	696.44 ± 331.36	1.15 ± 0.55	9.14 ± 5.57	0.27 ± 0.05
Co-exposure	129.77 ± 48.16 a	5.46 ± 3.31 ab	883.21 ± 261.15	1.47 ± 0.46	8.25 ± 3.45	0.29 ± 0.02

Based on Bayesian credibility intervals 95% (95% CrI), means followed by the same letters evidence that the posterior density does not overlap zero, therefore showing a statistically significant difference between groups. 1 = There is no difference between the treatments.

The behavior of young bees (3–4 days old) exposed to pesticides during the larval phase as well as that of those in the control groups did not differ significantly in relation to the mobile time, resting time, rotation and duration of high mobility (Table 3). The total distance traveled by the bees exposed to the fungicide in isolation as well as by the ones submitted to co-exposure was lower than in the control groups, as was the mean velocity (Table 3). The bees exposed to the insecticide in isolation did not differ from the control group in terms of total distance and mean velocity.

**Table 3:** Ingestion effects of pesticides-contaminated diet on the following behavioral variables (means ± standard error): mobile time, resting time, total moved distance, mean velocity, frequency of rotation and period of high mobility in honeybees (*A*. *mellifera*) at 3- to 4-days old.

Group	Mobile (s)^1^	Resting time (s)^1^	Total distance (cm)	Mean Velocity (cm/s)	Rotation (frequency)^1^	High mobility (s)^1^
Control	151.84 ± 31.03	1.53 ± 0.43	2165.51 ± 385.00 a	3.59 ± 0.64 a	17.00 ± 4.81	0.32 ± 0.03
Solvent control	179.26 ± 54.4	2.76 ± 1.05	2151.03 ± 694.49 a	3.55 ± 1.15 a	18.83 ± 6.68	0.37 ± 0.02
Clothianidin Insecticide	139.36 ± 42.72	5.30 ± 3.45	1840.24 ± 602.93 a	3.00 ± 0.97 a	15.83 ± 7.51	0.39 ± 0.03
Pyraclostrobin Fungicide	182.89 ± 33.67	0.81 ± 0.24	895.92 ± 151.40 b	1.47 ± 0.25 b	19.83 ± 3.07	0.32 ± 0.02
Co-exposure	141.77 ± 54.02	0.75 ± 0.35	886.82 ± 244.74 b	1.47 ± 0.40 b	9.17 ± 3.82	0.35 ± 0.06

Based on Bayesian credibility intervals 95% (95% CrI), means followed by the same letters evidence that the posterior density does not overlap zero, therefore showing a statistically significant difference between groups. 1 = There is no difference between the treatments.

### Survival analysis

**Figure 5:**
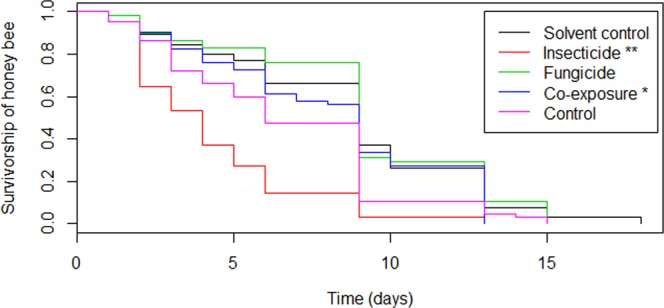
The survival pattern of young bees after emergence exposed in larval phase to pesticides Clothianidin insecticide and Pyraclostrobin fungicide, applied alone or combined. **p < 0.001, *p < 0.01.

Exposure to an insecticide during the larval phase affected the longevity of young bees (Fig. 5), reducing their life span (P < 0.001). Bees co-exposed to both pesticides during the larval phase also presented shorter longevity than those in the control group (P < 0.01), as shown in Fig. 5. No significant differences were observed regarding the longevity of bees in the control or solvent control (P > 0.9) groups and in bees exposed to the fungicide alone (P > 0.1).

There was a more accentuated slope on the survival curve for the clothianidin-exposed group when compared to the co-exposed group (Fig. 5), which was confirmed by the Median Lethal Time of bees exposed to the insecticide (Table 4).

**Table 4:** Ingestion effects of pesticides-contaminated diet on the median lethal time, in days, of honeybees (*A*. *mellifera*).

Treatment	*MLT* (*CL*)	χ^2^	*P*
Co-exposure	7.07 (5.93–8.15) a	88.70	<0.00001
Clothianidin Insecticide	3.11 (2.80–3.40) b	13.30	<0.00001
Control	7.85 (6.00–9.24) a	72.60	<0.00001
Pyraclostrobin Fungicide	6.91 (5.92–8.03) a	31.60	=0.00101
Solvent control	5.21 (4.34–6.02) a	48.20	<0.00001

*MLT*: Median Lethal Time. *CL*: Fiducial Confidence Limits (CL) with 95% of Probability. χ^2^: Chi-square. *P*: Probability.

Based on the overlap of 95% in fiducial limits, the median lethal time (MLT) values did not differ between the group submitted to co-exposure, to pyraclostrobin, the control and the solvent control (Table 4) groups. In a similar interpretation of the 95% fiducial limits overlap, the estimated MLT of the bees exposed to clothianidin insecticide was significantly different from that of other groups, indicating that honeybee exposure to clothianidin-contaminated diet during larval stage reduces the lifespan of adults.

## Discussion

Larval exposure to the clothianidin insecticide on its own and in combination with the pyraclostrobin fungicide did not alter larval mortality rates or post-embryonic development. The most significant effects were in the reduction of bee survival patterns after emergence, indicating a late effect of exposure during larval phase. Although the fungicide alone did not show effects on survival rates, the combination of pesticides and the fungicide on its own did affect the behavior of worker honeybees after emergence, reinforcing the late effect of larval exposure to these pesticides.

The reduction of distance traveled and mean velocity of young bees (3 to 4 days old) exposed to the fungicide and the ones submitted to co-exposure may be related to the stress generated by the metabolism caused by exposure to the fungicide during development, which can cause difficulty in food absorption in the midgut, where nutrients for the necessary energy supply are obtained so the bees can carry out all their activities^54^.

Pyraclostrobin acts in fungi on the inhibition of mitochondrial respiration by binding to the Qo site of cytochrome b^55^. According to Campbell *et al*.^36^, the formulation containing both fungicides - pyraclostrobin and boscalid - induced inhibition of mitochondrial function at 5ppm or higher concentration when applied directly to isolated thoracic mitochondria, but the exposure of forager honeybees to these fungicides did not indicate inhibition of oxygen consumption rates in mitochondria isolated from flight muscle of exposed bees. In the present study, the low dose of pyraclostrobin consumed per larva indicated behavioral changes in adults that may indirectly have been due to a possible decrease in the production of Adenosine Triphosphate (ATP) during development, so that there was a decrease in the locomotion activity of young bees. Thompson *et al*.^56^ suggest that the toxicity of the fungicide is highly dependent on its ingested dose by the honeybees. However, even at low doses, disturbances in larval development were observed in other classes of fungicide^57^.

Neonicotinoids have been shown to cause behavioral changes in adult bees, such as difficulty in returning to colonies and memory, orientation and communication impairment, as well as tremors and restlessness^53,58–65^. Clothianidin coming into contact with adult bees causes depolarization of Kenyon cells (neurons located in the pedunculated bodies of the brain) by binding to nicotinic acetylcholine receptors (nAChRs) and acting as a blocker of these cholinergic receptors in prolonged exposures, which may impair cognitive functions in bees^45,59,66^.

Larvae of Africanized *A*. *mellifera* exposed to the neonicotinoid thiamethoxam suffered premature cell death in their optic lobes^31^, which may compromise visual acuity and consequent disorientation in adult bees, causing them to process a smaller amount of visual information in the pedunculated bodies. Additionally, Friol *et al*.^17^ showed cellular damage in the midgut and tubules of Malpighi of honeybees, as well as in Kenyon cells in brain pedunculated bodies, mainly in the mitochondria, in newly emerged workers that had been exposed as larvae to 1 μg/L thiamethoxam, similar to that used in this study. Differently from what was expected, no behavioral changes were observed in honey bees exposed during larval phase to clothianidin, but these morphological and tissue changes and their consequences may be associated with the decreased longevity observed in the present study, since clothianidin is a metabolite of thiamethoxam.

The decreased longevity provoked by low doses of clothianidin is difficult to be detected in field conditions, making it hard to diagnose this post-emergence effect of larval exposure to pesticides. However, the late effect reported in the present study shows a possible reduction in the number and performance of worker bees in the hive, which may result in a rapid loss of the bee population. As in other studies with different species of bees, they were more tolerant to neonicotinoids in larval stages than as adults^10,67,68^.

A hydrophilic pesticide probably circulates in the hemolymph, possibly coming into contact with the organs during the larval phase, and it may be more easily excreted through the Malpighi tubules at the end of this phase, when defecation occurs^16^ and the excreta is released. If the pesticide is hydrophobic or partially hydrophobic, such as the clothianidin insecticide, with water solubility of 327 mg/L (20 °C)^69^, and the fungicide pyraclostrobin, with water solubility of 1,9 mg/L (20 °C), they accumulate in the fat body. When the fat body mobilizes the nutrients during the metamorphosis to supply the metabolic needs of the pupa, the chemical compound that was stored in the larva may also be mobilized and reach the hemolymph and, at that point, their molecules to begin to exert their effects on the organs. In the case of clothianidin, it would act in the bees’ brains during their pupal phase, which would compromise the morphophysiology of the pedunculated bodies^17,70^. This storage of neonicotinoid, including clothianidin, in the fat body of *A*. *mellifera*, was demonstrated by Feng *et al*.^69^. In the case of the fungicide, while it does not target a specific organ, it can interfere in the oxygen consumption rate and consequent ATP production of the mitochondria in general, pointing to the possibility that its effect may be diluted in the organism due to its its systemic action. This hypothesis needs to be tested in further studies.

Higher tolerance of larvae to insecticide exposure may be due to differences in gene expressions of nAChR subunits between the larval and adult phases, which begins to intensify in the pupal stage^71^. Moreover, in the beginning of larval development, there is a smaller amount of structures containing acetylcholine receptors in target organs, such as the Kenyon cells in brain pedunculated bodies^67^. Therefore, with a small number of target sites in the larval stage, the insecticide clothianidin in sublethal doses will show toxicity in later stages of bee development and after emergence.

Additionally, exposure of larvae to different doses of thiamethoxam, as observed by Tavares *et al*.^32^, generated an increase in acetylcholinesterase activity in pupae and newly emerged bees, reinforcing our hypothesis of neonicotinoid action from the metamorphorsis, when it would be mobilized from the storage organs to hemolymph. These authors also observed an increase in the activity level of the glutathione-S-transferase (GST) and the carboxylesterase enzymes in the pupa phase, indicating the activation of the detoxification process after the larval exposure to the pesticide.

Adult bees, under natural conditions, have a lower amount of GST than pupae^32,72^. As neonicotinoids bind to acetylcholine receptors in a stable manner, especially in the pupal stage, the neurotoxic effect probably remains in the newly emerged bee, which in turn has a lower capacity for detoxification by phase II enzymes (e.g., GST - glutathione transferases) in comparison to the pupa. This hypothesis might explain the decreased longevity of bees exposed or co-exposed to clothianidin in the larval phase when compared to the control group.

The decrease in the survival rates curve of bees exposed to the insecticide alone was more pronounced than in bees that were co-exposed to both pesticides. The combination of pyraclostrobin and clothianidin and its effects on the longevity of honey bee workers is unclear. Future studies on chemical interactions between these molecules may contribute to clarify the effects of this combination on bees.

Since fungicides of the strobilurin class can inhibit mitochondrial respiration in honey bees^35,36^, and seeing as it caused a reduction in the longevity of chronically exposed adult honeybees^38^, we conclude that the sublethal dose used in the present study was not sufficiently high to decrease the longevity of fungicide-exposed bees, although it was enough to affect their behavior.

In summary, co-exposure to the clothianidin insecticide and the pyraclostrobin fungicide did not show either an additive or synergistic effect of these pesticides at the realistic doses in which they are found in the food resources of larvae, according to the parameters evaluated in the present study. According to Thompson *et al*.^56^, insecticides and fungicides at low doses may cause a small increase in toxicity or lack of synergism.

In conclusion, larvae exposed to realistic, environmentally relevant doses of these pesticides, showed a late effect after emergence. Clothianidin, both in its isolated and combined forms, reduced longevity of emerged adults. On the other hand, pyraclostrobin reduced mean velocity and travelled distance of young adults, both in its isolated and combined forms. Thus, these changes could interfere in important worker tasks inside the hive and in the population dynamics of the colony.

## Method

### Chemicals

The chemicals used for toxicity test were clothianidin (CAS number 210880-92-5, ≥98% purity), pyraclostrobin (CAS number 175013-18-0, ≥98% purity) and dimethoate (CAS number 1219794-81-6, ≥98% purity). All of them were purchased from Sigma-Aldrich^TM^ St. Louis, Missouri, USA.

### Honeybee Larval Toxicity Test, Repeated Exposure to pesticides

The methodology followed the OECD n° 239^73^ protocol. First, instar larvae were collected from the comb of three different healthy colonies of Africanized *A*. *mellifera*, each one representing a replicate, and they were individually transferred into crystal polystyrene grafting cells which were previously sterilized. Each grafting cell, containing 20 μL of the standardized artificial diet in its interior, was placed at the bottom of 48 well-culture plates on a piece of dental cotton roll wetted with a sterilizing solution (0,2% w/v methylbenzethonium chloride) containing glycerol. The larval feed consisted of 50% w/w sugary solution (containing D-glucose, D-fructose and yeast extract) and 50% w/w royal jelly^74^.

The larvae were placed on 12 plates, which were separated into four blocks. Each block consisted of 3 plates, with each plate representing 1 colony (each colony representing a replicate). Each plate contained six experimental groups, each of which consist of the individual cell containing a larva. In this manner, the experimental group and its respective total number of larvae (number of larvae/plate *x* 4 blocks *x* 3 colonies) were: I - Control (n = 96), II - Solvent control (acetone) (n = 95), III - Clothianidin Insecticide (n = 95), IV - Pyraclostrobin Fungicide (n = 96), V - Combination (Insecticide + Fungicide) (n = 96) and VI - Dimethoate (n = 86), which is a reference chemical (positive control for larval mortality) according OECD n° 239^73^ due to its toxicity being well established for honey bees^75^.

The plates were placed into a hermetic desiccator that were kept in an incubator at a relative humidity of 95% ± 5% and temperature of 34 ± 2 °C, under dark conditions.

From the third (D3) to the sixth day (D6) after grafting, the test solution was mixed with the diet and supplied to each larva according to each experimental group. The concentrations used to prepare the diet with pesticides were based on realistic concentrations. For the clothianidin insecticide, this amount was similar to the maximum concentration found in pollen^76^ and bee bread^77^, i.e. 50 μg.L^−1^. For the pyraclostrobin fungicide, it was similar to the concentration found in pollen^22^, i.e. 5,000 μg.L^−1^. The dimethoate insecticide was used to validate the test at the concentration of 200 ng.μL^−1^ of the diet, adapted from OECD n° 239^73^.

Based on both the concentrations of the pesticides present in pollen and the estimated amount of pollen consumed by the honey bee larva during feeding phase, which was 5.4 mg^15,78^, the intake per larva was calculated. The concentration of pyraclostrobin in the diet offered to larvae was 169 μg.L^−1^, and so the daily volume of the diet provided per individual cell was used to calculate the dose ingested per larvae during feeding time (Intake for 4 days = 23.63 ng/larvae). The concentration of clothianidin in the diet offered to larvae was 1.69 μg.L^−1^, thus the intake for 4 days was 0.2364 ng/larvae. For the experimental group exposed simultaneously to both pesticides, the diet had the same concentration of the two chemical compounds applied in their isolated form, so that the intake during 4 days remained the same as that of the non-combined pesticides.

In the solvent-control group, the diet had the same volume of acetone as the one used for the treatments with pesticides, which did not exceed 2% of the diet’s final volume during the exposure period (from D3 to D6).

On the seventh day of bioassay (D7), the humidity inside the dessicator chamber and incubator was decreased to 80% ± 5%. On the fifteenth day (D15), the grafting cells with live pupae were transferred to plastic pots (9 cm × 9.2 cm) specific for the imago emergence. The individuals were evaluated until the death of the last of them, being fed with 50% (w/v) sucrose aqueous solution.

### Behavioral analysis by video tracking

Newly emerged bees (1 day old) and young bees (from 3 to 4 days old) were individually transferred to a Petri dish (9 × 1.5 cm). Each bee stayed for four minutes on the plate in order to acclimate. Then, a video recording of ten minutes per bee^68^ was carried out between 1–6 pm. For each experimental group, a sample of 7 bees that included all the blocks were submitted to filming according to their age, i.e., N = 7 per group, per age.

The videos were analyzed in the program Ethovision^79^, considering the following variables: mobile time, resting time, total moved distance, mean velocity, frequency of rotation and period of high mobility.

### Statistical analysis

We carried out all statistical analyzes using R (version 3.4.3; R Foundation for Statistical Computing, Vienna, AT, 2017). Two generalized linear models were fitted to proportion data. For the larval mortality and pupation rates we used a binomial model, and for pupal and emerging insect rates we used a quasibinomial model. An analysis of deviance was performed to assess whether the effects were non-significant (*P* = 0.05). The means were compared by means contrasts (95%) between the linear predictors of the fitted model. The goodness of fit was evaluated using half-normal plots with a simulated envelope^80^ employing the hnp package^81^.

Bayesian credible intervals of each behavioral variable of honey bees were calculated using the observations of the treatment, given *Y* = *y*, the interval [*a*, *b*] being (1-α) 100% Bayesian Credible Interval (*BCI*) for *X*, then if the posterior probability of *X* being in [*a*, *b*] is equal to (1-α), therefore, (1-α) = P (*a* ≤ *X* ≥ *b*|*Y* = *y*).

For the analysis of the credible intervals, we used 30,000 iterations using a Monte Carlo and Markov chains (MCMC) process with three strings for each parameter and with a burn-in of 5,000 samples. The convergence of the chains was checked by means of graphical analysis (data not included). The parameters were estimated using the *R2Openbugs* package^82^.

We conducted principal component analysis (PCA) to identify variation and ordination of the honey bees’ response to isolated and co-exposure to the clothianidin insecticide and the pyraclostrobin fungicide. The data was standardized by dividing the difference between each data point and the arithmetic mean of the variable of interest by the standard deviation of the same variable. The biplot contour area was defined by the eigenvectors values map as used by Malaquias *et al*.^83^. The components were selected for all PCA contingents of the eigenvalues in the correlation matrix according to the criterion by Kaiser^84^, based on the presence of components with eigenvalues greater than 1^85^.

Survival data was analyzed using the Cox proportional hazards regression model (survival package). We used the *R package ecotox* to conduct lethal time analysis of the time-mortality data to estimate the time survivorship of 50% of the tested insects (*MLT*) in each treatment, and the 95% fiducial limits of the *MLT*. We considered that two *MLT* values were significantly different only if their 95% fiducial limits did not overlap.
